# Efficacy of hyaluronic acid in the treatment of nasal inflammatory diseases: a systematic review and meta-analysis

**DOI:** 10.3389/fphar.2024.1350063

**Published:** 2024-02-07

**Authors:** Huixia Liu, Yue Chen, Huan Wang, Xinyi Luo, Dengpiao Xie, Qing Ji, Li Tian

**Affiliations:** ^1^ Hospital of Chengdu University of Traditional Chinese Medicine, Chengdu, Sichuan, China; ^2^ Chengdu Medical College, Chengdu, China; ^3^ Chengdu First People’s Hospital, Chengdu, Sichuan, China

**Keywords:** hyaluronic acid, sodium hyaluronate, allergic rhinitis, sinusitis, nasal inflammatory diseases, meta-analysis

## Abstract

**Background:** Hyaluronic acid (HA), the main component of the extracellular matrix, has the ability to promote tissue repair and regulate inflammation. It is used in otolaryngology as an adjuvant treatment to alleviate postoperative nasal symptoms. However, there is currently insufficient evidence demonstrating the therapeutic efficacy of HA for patients with nasal inflammatory diseases (NIDs). Therefore, this study aimed to evaluate the efficacy and safety of topical HA in the treatment of NID patients without receiving surgery.

**Methods:** In this meta-analysis, comprehensive searches were conducted in PubMed, Embase, the Cochrane Central Register of Controlled Trials, and Web of Science. Keywords searched included “hyaluronic acid,” “sinusitis,” “allergic rhinitis,” “rhinitis,” and “randomized controlled trials (RCTs).” The Cochrane Collaboration’s “Risk of Bias Assessment” tool was used to assess the quality of the included trials, and the meta-analysis was performed using the RevMan 5.3 and STATA 15 statistical software.

**Results:** A total of 11 articles and 825 participants were enrolled. For the primary outcomes, the pooled results revealed that HA significantly improves nasal obstruction (SMD, −0.53; 95% CI, −0.92 to −0.14; *p* = 0.008; and I^2^ = 79%) and rhinorrhea (SMD, −0.71; 95% CI, −1.27 to −0.15; *p* = 0.01; and I^2^ = 90%) in patients with NIDs. As for the secondary outcomes, the pooled results demonstrated that when compared with the control group, HA could significantly improve nasal endoscopic scores (*p* < 0.05), rhinitis scores (*p* < 0.05), rhinomanometry (*p* < 0.05), nasal neutrophils (*p* < 0.05), and mucociliary clearance (*p* < 0.05). However, no significant differences were observed between the two groups regarding nasal itching, sneezing, hyposmia, quality-of-life scores, and nasal eosinophils. For the risk of bias, 54.5% and 45.5% of trials had a low risk of bias in the randomization process and deviation of the intended intervention, respectively.

**Conclusion:** In the present study, the results reveal that HA might ameliorate symptoms of patients with NIDs. However, more clinical trials with larger participant cohorts are required to confirm this result.

**Systematic review registration number:** clinicaltrials.gov, identifier CRD42023414539.

## 1 Introduction

Nasal inflammatory diseases (NIDs) include a group of acute and chronic inflammations that occur in the nasal cavity and sinuses, which include allergic rhinitis (AR), non-allergic rhinitis (NAR), acute rhinosinusitis (ARS), chronic rhinosinusitis (CRS), atrophic rhinitis, etc. These types of diseases have been a common problem in the field of otolaryngology worldwide, with high incidence and recurrence rates, which are common causes for absence from work and visits to family doctors' offices. Epidemiological surveys have found that the global incidence of AR is 10%–20% (Brozek et al., 2010), and the incidence in some countries is as high as 40% (Bousquet et al., 2020). NAR affects over 200 million people worldwide (Bousquet et al., 2008). CRS affects 5%–12% of the population (Fokkens et al., 2020). In addition to causing nasal symptoms, NIDs can also lead to headaches, coughing, asthma, eye symptoms, and sleep disorders (Profita et al., 2010; Virchow et al., 2011; Rudmik, 2017; Kulthanan et al., 2018), severely affecting the quality of life of individuals and bringing a heavy economic burden to both society and individuals. Unfortunately, medical treatment for NIDs is often ineffective and prone to relapse, and a clear cure remains elusive. Guidelines recommend nasal irrigation as one of the complementary therapies for NIDs, which involves saline solution or hyaluronic acid (HA).

HA is a non-sulfated glycosaminoglycan composed of D-glucuronic acid and N-acetylglucosamine (Meyer, 1958). It is a component of the extracellular matrix and is found in the connective tissue, respiratory epithelium, nasal and tracheobronchial mucosa, airway secretions and glands, and serous cells (Choi et al., 2010). It plays an important role in cell signaling, leukocyte migration, cell adhesion, and biological remodeling (Macchi et al., 2013; Dicker et al., 2014; Pignataro et al., 2018; Zamboni et al., 2018). HA is a physiological component of nasal mucus, mainly produced by the goblet cells in the nasal mucosa (Tachibana and Morioka, 1990). In the nasal mucosa, HA can regulate vasomotor tone and glandular secretion (Gelardi et al., 2013a) and stimulate mucociliary clearance, playing an important role in mucosal host defense (Wolny et al., 2010). Furthermore, HA prevents bacterial adhesion, which exerts anti-infection and anti-biofilm effects *in vitro* (Drago et al., 2014). HA can also penetrate tissues, promote blood circulation, and improve intermediary metabolism and nutrient supply to tissues. As an endogenous anti-inflammatory molecule, HA can promote tissue regeneration and angiogenesis and inhibit inflammatory response during the wound healing process (Shaharudin and Aziz, 2016; Bai et al., 2020). The structure of HA lacks specific and allergenic properties, making it a highly safe molecule. Therefore, HA is widely used in various medical fields, such as plastic and cosmetic surgery, dermatology, ophthalmic surgery, otolaryngology, respiratory medicine, burn medicine, and such others (Ardizzoni et al., 2011). Some meta-analyses have demonstrated the efficacy and safety of topical HA in patients with endoscopic sinus surgery (Chen et al., 2017; Fong et al., 2017). This study aimed to assess the efficacy of HA as an adjuvant treatment for patients with NIDs without receiving surgery.

## 2 Materials and methods

### 2.1 Methods and search strategy

This study was performed based on the Preferred Reporting Items for Systematic Reviews and Meta-Analyses protocol statement guidelines. The protocol for this meta-analysis was registered on the PROSPERO platform with the registration number CRD42023414539. PubMed, Embase, the Cochrane Central Register of Controlled Trials, and Web of Science were searched up to March 2023. The search keywords included the Medical Subject Headings (MeSH) terms “Allergic Rhinitis,” “Sinusitis,” “Rhinitis,” “Hyaluronic Acid,” and “Randomized Controlled Trial.” English as the only language was searched. Taking the PubMed database as an example, the details of the search strategy and terms are listed in Supplementary Table S1. In addition, similar clinical studies and reviews were screened for potential studies.

### 2.2 Data sources and study selection

Two independent researchers scanned the titles and abstracts and downloaded the full text articles of the clinical trials. We independently assessed trials for eligibility and documented reasons for exclusion. If there were differences in opinion, we resolved them by consensus among the researchers. Finally, we reviewed the full text articles of the selected randomized controlled trials (RCTs).

### 2.3 Inclusion and exclusion criteria

The inclusion criteria were that the (1) study should be a RCT; (2) patients diagnosed with NID such as AR or CRS; (3) intervention should include HA; and (4) study should include primary or secondary outcomes. The primary outcomes were nasal obstruction and rhinorrhea. The secondary outcomes were nasal itching, sneezing, hyposmia, rhinitis scores, nasal pressure, nasal endoscopy scores, mucociliary clearance, nasal eosinophil count, and neutrophil count. The trials were excluded if they were (1) animal or cellular studies; (2) non-comparative studies; (3) non-RCTs; (4) previous nasal surgery due to NIDs; (5) studies without available data; (6) repeat published trials; (7) case reports, comments, letters, reviews, retrospective studies; and (8) ongoing trials.

### 2.4 Data extraction and quality assessment

The study characteristics (author’s first name, year of publication, country, and study duration), baseline characteristics of patients (age, gender, and type of NID), intervention strategy, outcomes, and adverse events were extracted. The Cochrane Collaboration’s “Risk of Bias Assessment” tool was used to assess the quality of the included trials. It contained the randomization process, deviation from the intended intervention, missing outcome data, measurement of the outcome, selection of the reported result, and overall risk of bias. Finally, three kinds of quality evaluations were made for literature, namely “low,” “high,” and “uncertain” risk of bias. Two independent reviewers assessed the risk of bias. Any discrepancies were resolved by the third author. The corresponding author was responsible for contacting the authors of the trials for missing information and unpublished data.

### 2.5 Data synthesis and analysis

The pooled result was shown as the standardized mean difference (SMD) and 95% confidence interval (CI). Heterogeneity was reported as I^2^, and I^2^ values <25%, between 25% and 50%, and >50% were regarded as low, moderate, and high heterogeneity, respectively (Higgins et al., 2003). A random model was applied in this study. We performed subgroup analyses to assess certain factors that influence the pooled results and the sources of heterogeneity. Subgroup analyses were performed based on sinusitis or non-sinusitis.

If the outcome included more than 10 trials, we performed a funnel plot asymmetry test to assess the publication bias. Sensitivity analysis was performed by the leave-one-out method to assess the stability of the primary or secondary outcome. The Cochrane ReviewManager 5.3 (Oxford, United Kingdom) and STATA 15 software were used for the present meta-analysis. *p*-value < 0.05 was considered statistically significant.

## 3 Results

### 3.1 Study selection and characteristics

**FIGURE 1:**
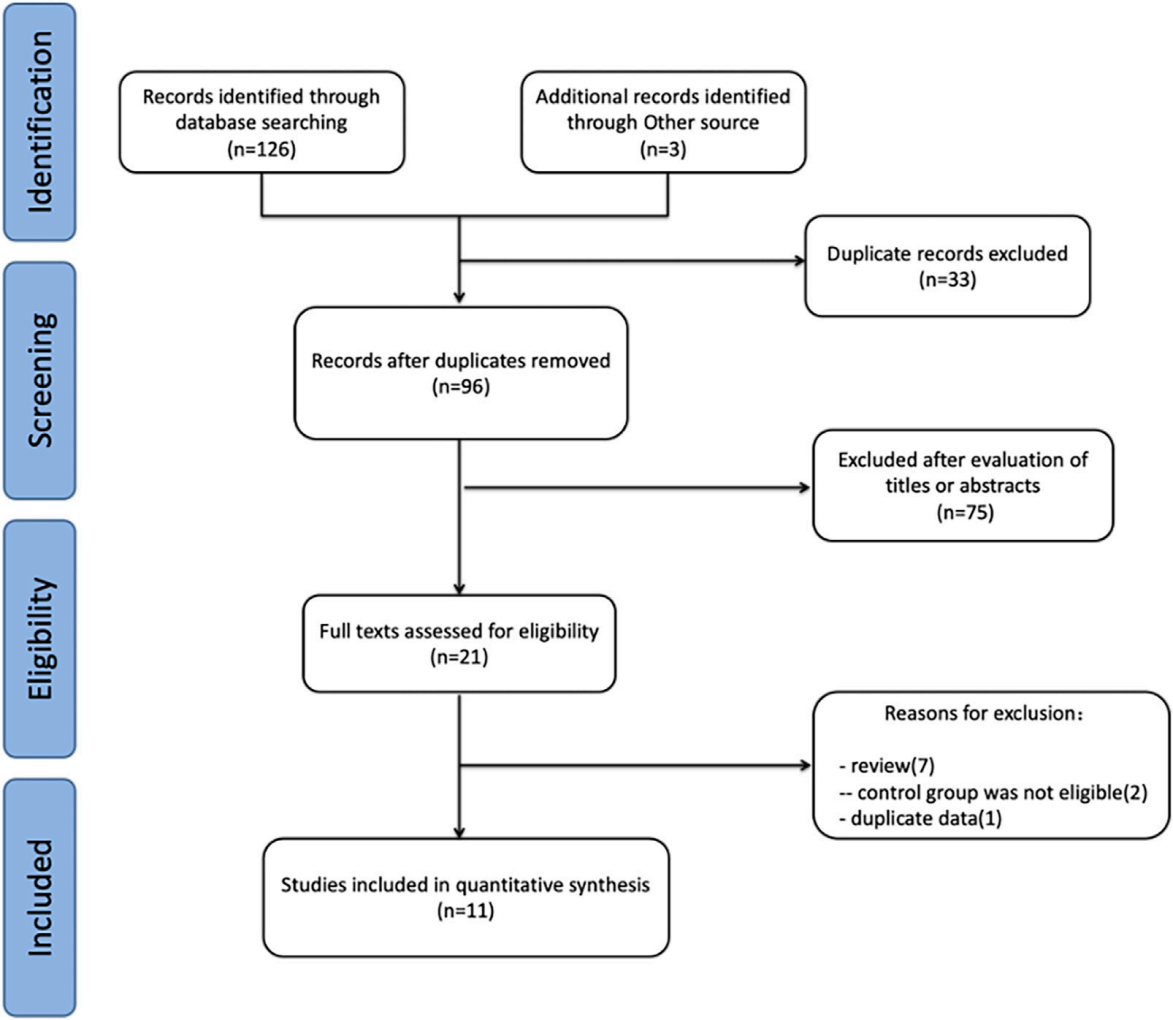
Study selection flow for the present meta-analysis review.

The initial search identified 129 relevant studies: 38 from PubMed, 26 from Embase, 29 from the Web of Science, 33 from the Cochrane Library, and three articles from other sources. After excluding duplicate articles and further evaluating the full text of the remaining articles, 11 articles (Gelardi et al., 2013b; Casale et al., 2014; Cassandro et al., 2015; Cantone and Iengo, 2016; Gelardi et al., 2016; Ciofalo et al., 2017; Favilli et al., 2019; Savietto et al., 2020; Thieme et al., 2020; Ocak et al., 2021; Ercan et al., 2022) were finally included in this meta-analysis. Ercan et al. (2022) and Cassandro et al. (2015) had two treatment groups, which were identified as Ercan a and Ercan b and Cassandro a and Cassandro b, respectively. The flow diagram of the study selection is presented in Figure 1.

### 3.2 Participants

A total of 11 studies (Gelardi et al., 2013b; Casale et al., 2014; Cassandro et al., 2015; Cantone and Iengo, 2016; Gelardi et al., 2016; Ciofalo et al., 2017; Favilli et al., 2019; Savietto et al., 2020; Thieme et al., 2020; Ocak et al., 2021; Ercan et al., 2022) and 825 participants met the inclusion criteria. Four studies (Casale et al., 2014; Cassandro et al., 2015; Cantone and Iengo, 2016; Savietto et al., 2020) were CRS, one study (Ciofalo et al., 2017) was ARS, two studies (Ocak et al., 2021; Ercan et al., 2022) were AR, one study (Favilli et al., 2019) was pregnancy rhinitis (PR), one study (Thieme et al., 2020) was rhinitis sicca, and two studies (Gelardi et al., 2013b; Gelardi et al., 2016) included multiple rhinitis. Eight studies (Gelardi et al., 2013b; Casale et al., 2014; Cassandro et al., 2015; Cantone and Iengo, 2016; Gelardi et al., 2016; Ciofalo et al., 2017; Favilli et al., 2019; Savietto et al., 2020) were from Italy, two studies (Ocak et al., 2021; Ercan et al., 2022) were from Turkey, and one study (Thieme et al., 2020) was from Germany. The duration of the intervention administration varied between studies and ranged from 4 weeks to 3 months (Gelardi et al., 2013b; Casale et al., 2014; Cassandro et al., 2015; Cantone and Iengo, 2016; Gelardi et al., 2016; Ciofalo et al., 2017; Savietto et al., 2020; Thieme et al., 2020; Ocak et al., 2021; Ercan et al., 2022). Due to the particularity of pregnant patients (Favilli et al., 2019), the end time of medication was determined according to the time of delivery. The characteristics of these 11 studies are shown in Table 1.

**TABLE 1:** Basic characteristics of included studies.

Source	Country	Type of patient	Duration	Intervention			Control			Outcomes	Adverse event
				Intervention method (dose)	Population (male)	Mean age	Intervention method (dose)	Population (male)	Mean age		
Cantone et al. (2016)	Italy	CRSwNP	3 months	Mometasone furoate nasal spray (200 μg, once daily)	40	56.9 ± 5.6^α^	Mometasone furoate nasal spray (200 μg, once daily)	40	56.8 ± 4.4^α^	Nasal congestion, rhinorrhea, nasal endoscopy scoring, and quality of life	No adverse reactions
				SH plus saline solution (9 mg, twice daily)			Saline solution (5 ml, twice daily)				
Casale et al. (2014)	Italy	CRS	3 months	SH plus saline solution (9 mg, twice/day)	21 (13)	44 (30–63)^β^	Saline solution (5 ml, twice/day)	18 (10)	38 (34–58)^β^	Rhinitis	No adverse reactions
Cassandro et al. (2015)	Italy	CRSwNP	3 months	SH plus saline (9 mg, twice daily)	20 (12)	38.75 ± 13.08^α^	Saline (5 ml, twice daily)	20 (11)	38.6 ± 13.06^α^	Rhinitis, mucociliary clearance, nasal endoscopy scoring, and rhinomanometry	Headache, throat irritation, upper respiratory infection, epistaxis, and nasal burning
				Mometasone furoate nasal spray (200 μg, twice daily)	20 (12)	38.85 ± 13.31^α^	Mometasone furoate nasal spray (200 μg, twice daily)	20 (10)	38.4 ± 12.7^α^		
				SH (9 mg, twice daily)							
Ciofalo et al. (2017)	Italy	ARS	30 days	Levofloxacin (500 mg, 10 days)	24 (12)	44 (38–50)*	Levofloxacin (500 mg, 10 days)	24 (14)	43 (35–55)*	Nasal congestion, rhinorrhea, eosinophils, neutrophils, mucociliary clearance, and hyposmia	Not reported
				Prednisone (50 mg, 8 days; 25 mg, 4 days; and 12.5 mg, 4 days)			Prednisone (50 mg, 8 days; 25 mg, 4 days; and 12.5 mg, 4 days)				
				SH plus saline solution (6 ml, twice daily)			Saline solution (6 ml, twice daily)				
Ercan et al. (2022)	Turkey	AR in children	28 days	Nasal fluticasone furoate (1 puff/nostril, once daily)	26 (18)	8.38 ± 1.89^α^	Nasal fluticasone furoate (1 puff/nostril, once daily)	24 (12)	8.5 ± 1.31^α^	Nasal congestion, rhinorrhea, rhinitis, itching, sneezing, eosinophils, quality of life, and rhinomanometry	Nasal irritation and burning sensation
				SH (twice daily)			Saline solution (twice daily)				
				Nasal fluticasone furoate (1 puff/nostril, once daily)	26 (18)	8.38 ± 1.89^α^	Nasal fluticasone furoate (1 puff/nostril, once daily)	26 (18)	8.69 ± 1.7^α^		
				SH (twice daily)							
Favilli et al. (2019)	Italy	Pregnancy rhinitis	Until delivery	SH (9 mg/vial; 2 vials daily for 14 days, followed by 15 days of interruption of therapy; subsequently 1 vial daily for 10 and 15 days of interruption of therapy; and lastly 1 vial daily for 10 days)	28	31.6 ± 5.5^α^	Did not receive any treatment	27	28.1 ± 4.8^α^	Rhinorrhea	No adverse reactions
Gelardi et al. (2013a)	Italy	AR and vasomotor rhinitis	30 days	Mometasone furoate nasal spray (50 μg/spray, 2 sprays/nostril once daily)	39 (23)	21–63^β^	Mometasone furoate nasal spray (50 μg/spray, 2 sprays/nostril once daily)	39 (21)	22–61^β^	Nasal congestion, rhinorrhea, eosinophils, and neutrophils	Not reported
				Desloratadine (5 mg, once daily)			Desloratadine (5 mg, once daily)				
				SH (9 mg, twice daily)			Sodium chloride (6 ml, twice daily)				
Gelardi et al. (2016)	Italy	AR, NAR, and MR	4 weeks	Intranasal mometasone furoate (1 puff/nostril, twice daily)	48	Not reported	Intranasal mometasone furoate (1 puff/nostril, twice daily)	41	Not reported	Nasal congestion, rhinorrhea, itching, sneezing, and hyposmia	No adverse reactions
				Rupatadine fumarate (1 tablet daily)			Rupatadine fumarate (1 tablet daily)				
				Isotonic saline solution (1 puff/nostril, twice daily)			Isotonic saline solution (1 puff/nostril, twice daily)				
				SH (1 cm per nostril in the afternoon)							
Ocak et al. (2021)	Turkey	AR	30 days	Triamcinolone acetonide sprays (256 μg/day, 1 puff/nostril, once daily)	32 (14)	34 (18–68) ^β^	Triamcinolone acetonide sprays (256 μg daily, 1 puff/nostril, once daily)	33 (13)	36 (18–61) ^β^	Mucociliary clearance	No adverse reactions
				Desloratadine (5 mg, once daily)			Desloratadine (5 mg, once daily)				
				SH (9 mg, twice daily)			Isotonic saline (9 mg, twice daily)				
Savietto et al. (2020)	Italy	CRSsNP	30 days	SH (5 mg, twice daily)	15	Not reported	Isotonic saline solution (5 mg, twice daily)	15	Not reported	Nasal congestion, rhinorrhea, eosinophils, neutrophils, nasal endoscopy scoring, quality of life, and hyposmia	No adverse reactions
Thieme et al. (2020)	Germany	Dry nose symptoms	4 weeks	SH (1–2 sprays/nostril)/Hyaluronic acid plus dexpanthenol (1–2 sprays/nostril)	79 (41)/80 (25)	54.15 ± 17.03^α^/50.60 ± 18.98^α^	Isotonic saline (1–2 sprays/nostril)	80 (31)	50.27 ± 19.7^α^	Nasal Congestion, Rhinorrhea, Rhinitis, Itching, Sneezing, Hyposmia	Cephalgia

CRS, chronic rhinosinusitis; CRSwNP, chronic rhinosinusitis with nasal polyposis; CRSsNP, chronic rhinosinusitis without nasal polyposis; ARS, acute rhinosinusitis; NAR, non-allergic rhinitis; MR, mixed rhinitis; SH, sodium hyaluronate; α, mean age ± SD; β, mean age (range); *, median (IQR).

### 3.3 Intervention

Participants in the study were randomly divided into the treatment and control groups. The interven in the control group was normal saline or active treatment, and the intervention in the HA group was HA or the addition of HA to that in the control group. One study did not receive any intervention in the control group (Favilli et al., 2019). One study used cream HA (Gelardi et al., 2016), and the remaining 10 trials used liquid HA. The dosage of HA was different in different studies. There were six trials (Gelardi et al., 2013b; Casale et al., 2014; Cassandro et al., 2015; Cantone and Iengo, 2016; Favilli et al., 2019; Ocak et al., 2021) with a dose of 9 mg, one trial (Ciofalo et al., 2017) with a dose of 6 ml, one trial (Savietto et al., 2020) with a dose of 5 mg, one trial (Thieme et al., 2020) with a dose of 1–2 sprays/nostril, and one trial (Gelardi et al., 2016) with a 1 cm dose of intranasal cream in each nostril, and one trial (Ercan et al., 2022) did not mention the dose.

### 3.4 Outcomes

#### 3.4.1 Primary outcomes: nasal obstruction and rhinorrhea

**FIGURE 2:**
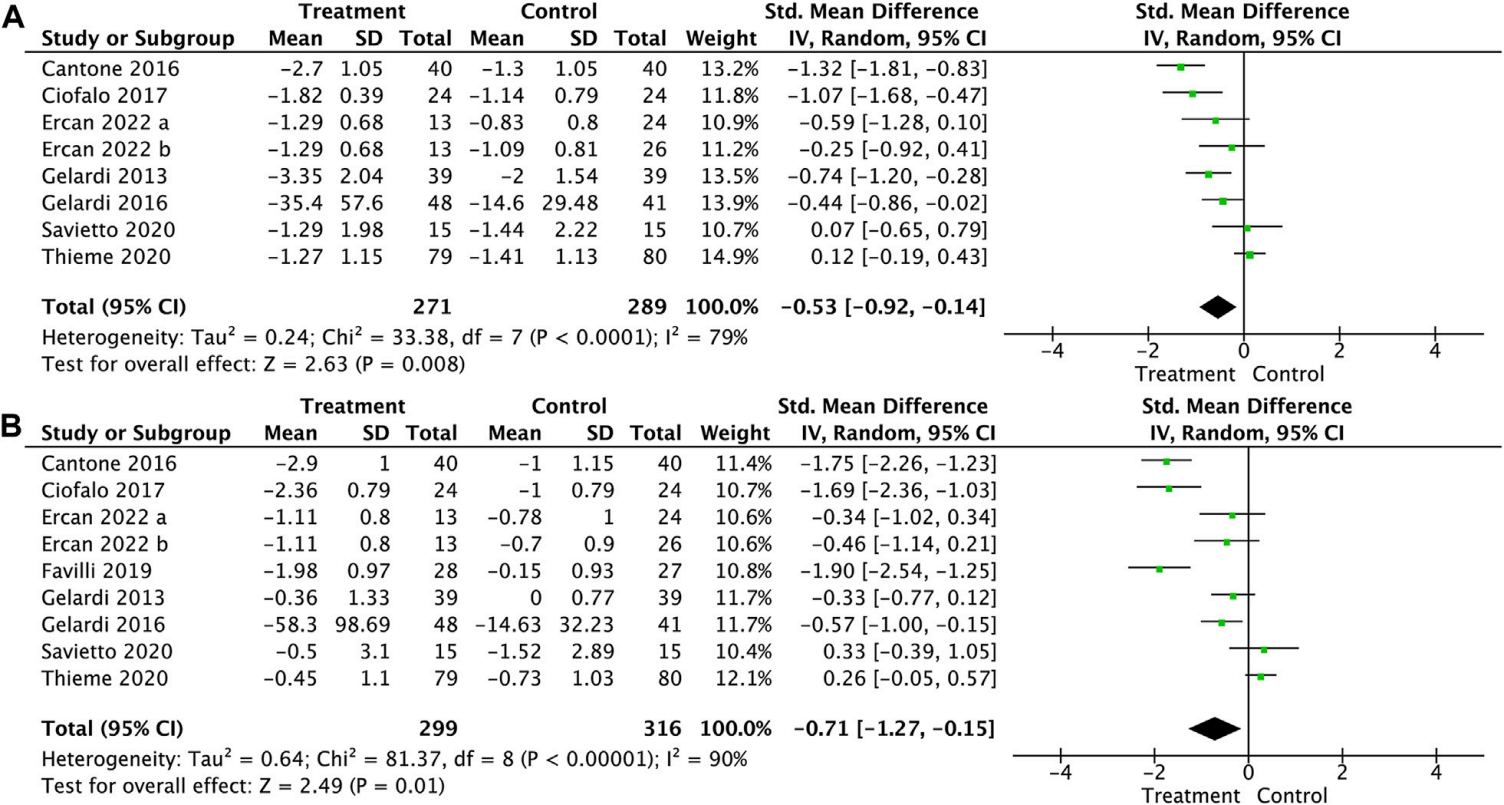
Forest plot for the effect of HA on nasal obstruction **(A)** and rhinorrhea **(B)**.

Nasal obstruction was included in seven trials (Gelardi et al., 2013b; Cantone and Iengo, 2016; Gelardi et al., 2016; Ciofalo et al., 2017; Savietto et al., 2020; Thieme et al., 2020; Ercan et al., 2022) for the meta-analysis. The result showed that HA significantly reduced nasal obstruction symptoms (SMD, −0.53; 95% CI, −0.92 to −0.14; *p* = 0.008; and I^2^ = 79%, Figure 2 A).

Rhinorrhea was measured in eight trials (Gelardi et al., 2013b; Cantone and Iengo, 2016; Gelardi et al., 2016; Ciofalo et al., 2017; Favilli et al., 2019; Savietto et al., 2020; Thieme et al., 2020; Ercan et al., 2022). Pooled results showed that HA significantly relieved rhinorrhea symptoms (SMD, −0.71; 95% CI, −1.27 to −0.15; *p* = 0.01; and I^2^ = 90%, Figure 2 B).

#### 3.4.2 Secondary outcomes: nasal endoscopic scores, rhinitis scores, rhinomanometry, neutrophils, mucociliary clearance, nasal itching, sneezing, quality-of-life scores, eosinophils, and hyposmia

Of the 11 trials, nasal endoscopic scores were included in three trials (Cassandro et al., 2015; Cantone and Iengo, 2016; Savietto et al., 2020), rhinitis scores were included in four trials (Casale et al., 2014; Cassandro et al., 2015; Thieme et al., 2020; Ercan et al., 2022), rhinomanometry was included in two trials (Cassandro et al., 2015; Ercan et al., 2022), neutrophils were included in three trials (Gelardi et al., 2013b; Ciofalo et al., 2017; Savietto et al., 2020), mucociliary clearance was included in three trials (Cassandro et al., 2015; Ciofalo et al., 2017; Ocak et al., 2021), nasal itching was included in three trials (Gelardi et al., 2016; Thieme et al., 2020; Ercan et al., 2022), sneezing was included in three trials (Gelardi et al., 2016; Thieme et al., 2020; Ercan et al., 2022), hyposmia was included in four trials (Gelardi et al., 2016; Ciofalo et al., 2017; Savietto et al., 2020; Thieme et al., 2020), quality-of-life scores were included in three trials (Cantone and Iengo, 2016; Savietto et al., 2020; Ercan et al., 2022), and eosinophils were included in four trials (Gelardi et al., 2013b; Ciofalo et al., 2017; Savietto et al., 2020; Ercan et al., 2022).

**FIGURE 3:**
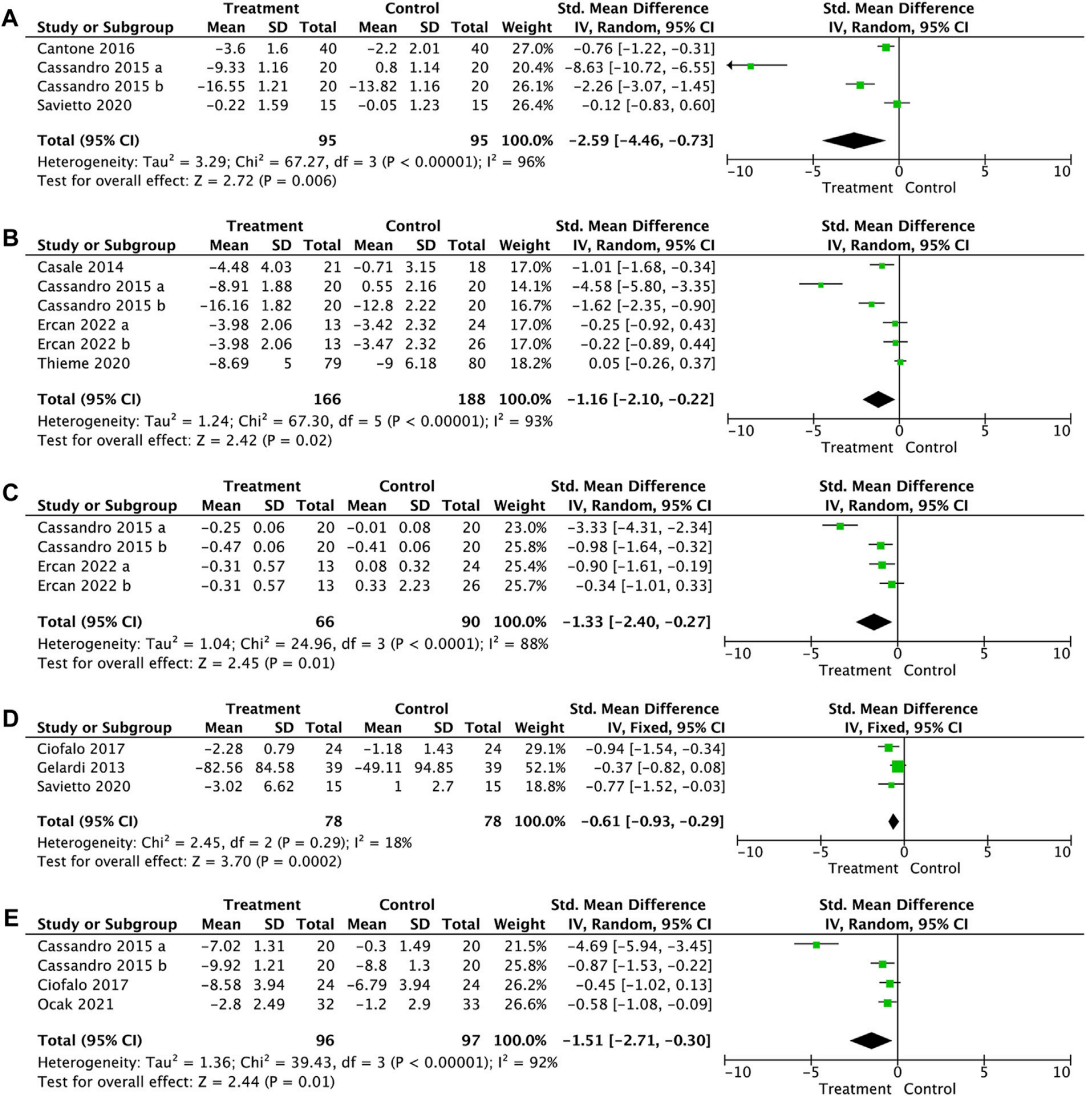
Forest plot for the effect of HA on nasal endoscopic score **(A)**, rhinitis score **(B)**, rhinomanometry **(C)**, nasal neutrophils **(D)**, and mucociliary clearance **(E)**.

The pooled results showed that when compared with the control group, HA could significantly improve the nasal endoscopic score (SMD, −2.59; 95% CI, −4.46 to −0.73; *p* = 0.006; and I^2^ = 96%, Figure 3 A), the rhinitis score (SMD, −1.16; 95% CI, −2.10 to −0.22; *p* = 0.02; and I^2^ = 93%, Figure 3 B), rhinomanometry (SMD, −1.33; 95% CI, −2.40 to −0.14; *p* = 0.01; and I^2^ = 88%, Figure 3 C), nasal neutrophils (SMD, −0.61; 95% CI, −0.93 to −0.29; *p* = 0.0002; and I^2^ = 18%, Figure 3 D), and mucociliary clearance (SMD, −1.51; 95% CI, −2.71 to −0.30; *p* = 0.01; and I^2^ = 92%, Figure 3 E). No significant differences were observed between the two groups in nasal itching (SMD, 0.06; 95% CI, −0.16 to 0.29; *p* = 0.57; and I^2^ = 0%, Supplementary Figure S1 A), sneezing (SMD, −0.14; 95% CI, −0.36 to 0.08; *p* = 0.23; and I^2^ = 0%, Supplementary Figure S1 B), hyposmia (SMD, −0.04; 95% CI, −0.26 to 0.17; *p* = 0.70; and I^2^ = 27%, Supplementary Figure S1 C), quality-of life-score (SMD, −0.46; 95% CI, −0.99 to 0.08; *p* = 0.10; and I^2^ = 66%, Supplementary Figure S1 D), and nasal eosinophils (SMD, 0.04; 95% CI, −0.25 to 0.34; *p* = 0.78; and I^2^ = 24%, Supplementary Figure S1 E).

#### 3.4.3 Subgroup analysis

The subgroup analysis was performed by dividing the patients into the sinusitis or non-sinusitis groups. The subgroup analysis showed that HA could significantly reduce the symptoms of nasal obstruction and rhinorrhea in patients with sinusitis or non-sinusitis (Supplementary Figure S2).

#### 3.4.4 Adverse events

Of the 11 studies included, six studies (Casale et al., 2014; Cantone and Iengo, 2016; Gelardi et al., 2016; Favilli et al., 2019; Savietto et al., 2020; Ocak et al., 2021) reported no adverse events, three studies (Gelardi et al., 2013b; Ciofalo et al., 2017; Thieme et al., 2020) did not mention adverse events, and two studies (Cassandro et al., 2015; Ercan et al., 2022) reported adverse events. These studies (Cassandro et al., 2015; Ercan et al., 2022) reported adverse events including nasal burning, headache, throat irritation, upper respiratory tract infection, epistaxis, and nasal irritation. One patient reported thrice about the occurrence of cephalgia in hyaluronic acid plus dexpanthenol group, rated as possibly related to the application of the nasal spray (Thieme et al., 2020). One trial (Cassandro et al., 2015) reported no difference in the incidence of adverse events between the HA and control groups, and the other trial (Ercan et al., 2022) showed only mild adverse events in the control group.

### 3.5 Sensitivity analysis and publication bias

Sensitivity analyses were performed on the primary outcome measure by omitting one study at a time. The sensitivity analysis results showed that the pooled result and heterogeneity had no significant changes in nasal obstruction and rhinorrhea (Supplementary Figure S3).

### 3.6 Risk-of-Bias Assessment

**FIGURE 4:**
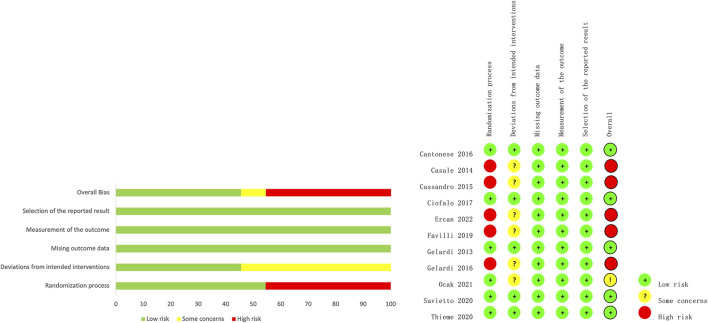
Summary of the risk bias of the included studies.

In total, 54.5% of the included trials had a low risk of bias in terms of the randomization process, 45.5% had a low risk of bias in terms of deviation from the intended intervention, and all trials had a low risk of bias in terms of the lack of outcome data, measurement of outcomes, and choice of reporting outcomes. Figure 4 shows the results for each risk of bias item for each study. The details of the risk of bias assessment are shown in Supplementary Table S2.

## 4 Discussion

In this meta-analysis, we identified 11 relevant studies involving 825 participants. The pooled results showed that HA significantly improved nasal congestion, rhinorrhea, nasal endoscopy scores, rhinitis scores, rhinomanometry, neutrophil infiltration, and mucociliary clearance. However, no significant differences were observed between the two groups in terms of nasal itching, sneezing, hyposmia, and quality-of-life score. HA does not increase side effects and is well tolerated by all patients.

HA has been reported to be safe and well tolerated and is recommended as one of the drugs for nasal irrigation administrations. HA has been recommended as an adjunct treatment following sinus surgery. Fong et al. (2017) conducted a meta-analysis, which included 13 RCTs and 501 patients, and pooled results showed that HA can limit adhesion formation after endoscopic sinus surgery (ESS) in patients with CRS. Another meta-analysis revealed that HA can significantly improve reepithelization and reduce edema after ESS (Chen et al., 2017). In our meta-analysis, we found that HA can also significantly improve clinical symptoms in patients with NIDs. These results have indicated that HA is suitable for not only patients with sinus surgery but also patients with NIDs without surgery. The treatment effect of HA might be associated with its biological properties, such as mucosal repair and healing and regulation of inflammatory responses. In addition, topical application of HA creates a thin protective layer on the surface of the nasal mucosa, which helps prevent allergens and irritants from adhering to the lining of the nasal cavity, thereby reducing the occurrence of allergic or irritant reactions. In the present study, we only study the effect of HA on neutrophils and eosinophils as inflammatory cells; however, the effect of HA on other surrogate markers of nasal inflammation remains unknown. Therefore, the effect of HA on other inflammatory markers has to be explored, such as fraction of exhaled nitric oxide, interleukin, and tumor necrosis factor (Galli et al., 2012; Ventura et al., 2013).

NID is a general term for nasal cavity and sinus inflammation. In addition to common CRS and AR, rhinitis medicamentosa (RM) and PR also belong to NID. Decongestants are common drugs for the treatment of NIDs. However, the long-term use of decongestants can lead to inferior turbinate vasodilation, mucosal edema, and decreased efficacy. HA has lubrication and tissue repair effects. Casale et al. (2017) found that HA regulates vasomotor and glandular secretion in the nasal mucosa, stimulates cilia to remove foreign matter and retain enzymes, promotes mucosal host defense, and has a good curative effect on RM. PR is induced by pregnancy and is often ignored by patients. PR is often accompanied by nasal obstruction, snoring, day and night fatigue, the inability to concentrate, headaches, thirst, etc. Snoring is an independent risk factor for hypertension, diabetes, and preeclampsia (Kowall et al., 1989; Feinsilver and Hertz, 1992; Franklin et al., 2000; Ursavas et al., 2008). Otorhinolaryngologists have to be very cautious about medication on PR, and most drugs used for PR are contraindicated. Due to the high safety of HA use, it can temporarily replace medicines used to relieve symptoms. Favilli et al. (2019) conducted a study of HA on rhinitis during pregnancy, and the results showed that HA could improve the symptoms of rhinitis during pregnancy without harming the health of the fetus. This provides a new idea for the relief of symptoms in patients with rhinitis during pregnancy.

In recent years, there have been more and more studies on the effect of HA in otolaryngology—head and neck surgery, which includes tympanic membrane perforation, middle ear surgery, vocal cord surgery, tracheal wounds, and nasal cavity surgery. Most results support the therapeutic effect of HA as adjuvant therapy, and this meta-analysis also concludes that HA can improve symptoms of NIDs.

This systematic review and meta-analysis had some limitations. First, the sample size of some included RCTs was small. Second, we observed high heterogeneity in primary outcomes. However, the risk of bias for randomization and deviation from the intended intervention was low only in approximately 50% of the trials, indicating that high heterogeneity comes from other factors, such as difference in duration of treatment, doses of HA, or severity of diseases. We further conducted subgroup analyses based on the sinusitis or non-sinusitis groups, but heterogeneity was not significantly reduced. We could not use more meaningful subgroups to reduce heterogeneity due to the limited trials. Third, we included disease groups rather than individual diseases, which is a source of clinical heterogeneity. Fourth, eight out of 11 trials are from the same country, which might increase the risk of bias and exaggerate the treatment effect.

In the present study, the results showed that HA might ameliorate the symptoms of patients with NIDs. However, more clinical trials with larger participant cohorts are required to confirm this result.

## Data Availability

The original contributions presented in the study are included in the article/Supplementary Material; further inquiries can be directed to the corresponding authors.

## Abbreviations

HA, Hyaluronic acid; NIDs, nasal inflammatory diseases; RCTs, randomized controlled trials; AR, allergic rhinitis; NAR, non-allergic rhinitis; ARS, acute rhinosinusitis; CRS, chronic rhinosinusitis; RM, rhinitis medicamentosa; PR, pregnancy rhinitis; MeSH, Medical Subject Headings; SMD, standardized mean difference; CI, confidence interval; ESS, endoscopic sinus surgery.
