# Corrigendum: Efficacy of hyaluronic acid in the treatment of nasal inflammatory diseases: a systematic review and meta-analysis

**DOI:** 10.3389/fphar.2024.1404434

**Published:** 2024-11-08

**Authors:** Huixia Liu, Yue Chen, Huan Wang, Xinyi Luo, Dengpiao Xie, Qing Ji, Li Tian

**Affiliations:** ^1^ Hospital of Chengdu University of Traditional Chinese Medicine, Chengdu, Sichuan, China; ^2^ Chengdu Medical College, Chengdu, China; ^3^ Chengdu First People’s Hospital, Chengdu, Sichuan, China

**Keywords:** hyaluronic acid, sodium hyaluronate, allergic rhinitis, sinusitis, nasal inflammatory diseases, meta-analysis

In the published article, there was an error in Table 1 as published. The authors found that the occurrence of cephalgia was possibly related the use of nasal spray (Thieme et al., 2020), however, this was omitted in the meta-analysis. The final row of Table 1 (source Thieme et al., 2020) has been corrected to reflect this. The corrected Table 1 and its caption appear below.

**TABLE 1:** Basic characteristics of included studies.

Source	Country	Type of patients	Duration	Intervention			Control Intervention			Outcomes	Adverse event
				Intervention method (Dose)	Population (Male)	Mean age	method (Dose)	Population (Male)	Mean age		
**Cantone et al., 2016**	Italy	CRSwNP	3 months	Mometasone furoate nasal spray (200μg, once daily) SH plus saline solution (9mg, twice daily)	40	56.9 ± 5.6α	Mometasone furoate nasal spray (200μg, once daily) Saline solution (5mL, twice daily)	40	56.8 ± 4.4α	Nasal Congestion, Rhinorrhea, Nasal Endoscopy Scoring, Quality of life	No adverse reactions
**Casale et al., 2014**	Italy	CRS	3 months	SH plus saline solution (9mg, twice/day)	21 (13)	44 (30–63) β	Saline solution (5mL, twice/day)	18 (10)	38 (34–58) β	Rhinitis	No adverse reactions
**Cassandro et al., 2015**	Italy	CRSwNP	3 months	SH plus saline (9mg, twice daily)	20 (12)	38.75 ± 13.08α	Saline (5mL, twice daily)	20 (11)	38.6 ± 13.06α	Rhinitis, Mucociliary clearance, Nasal Endoscopy Scoring, Rhinomanometry	Headache, Throat irritation, Upper respiratory infection, Epistaxis, Nasal burning
				Mometasone furoate nasal sprays (200μg, twice daily) SH (9mg, twice daily)	20 (12)	38.85 ± 13.31α	Mometasone furoate nasal sprays (200μg, twice daily)	20 (10)	38.4 ± 12.7α		
**Ciofalo et al., 2017**	Italy	ARS	30 days	Levofloxacin (500mg, 10days) Prednisone (50mg, 8days; 25mg, 4days; 12.5mg, 4days) SH plus saline solution (6mL, twice daily)	24 (12)	44 (38–50) *	Levofloxacin (500mg, 10days) Prednisone (50mg, 8days; 25mg, 4days; 12.5mg, 4days) Saline solution (6mL, twice daily)	24 (14)	43 (35–55) *	Nasal Congestion, Rhinorrhea, Eosinophils, Neutrophils, mucociliary clearance, Hyposmia	Not reported
**Ercan et al., 2022**	Turkey	AR in children	28 days	Nasal fluticasone furoate (1 puff/nostril, once daily) SH (twice daily)	26 (18)	8.38 ± 1.89α	Nasal fluticasone furoate (1 puff/nostril, once daily) Saline solution (twice daily)	24 (12)	8.5 ± 1.31α	Nasal Congestion, Rhinorrhea, Rhinitis, Itching, Sneezing, Eosinophils, Quality of life, Rhinomanometry	Nasal irritation and burning sensation
				Nasal fluticasone furoate (1 puff/nostril, once daily) SH (twice daily)	26 (18)	8.38 ± 1.89α	Nasal fluticasone furoate (1 puff/nostril, once daily)	26 (18)	8.69 ± 1.7α		
**Favilli et al., 2019**	Italy	Pregnancy Rhinitis	until delivery	SH (9mg/vial; 2 vials daily for 14 days, followed by 15 days of interruption of therapy; subsequently 1 vial daily for 10 and 15 days of interruption of therapy; lastly 1 vial daily for 10 days)	28	31.6 ± 5.5α	Not receive any treatment	27	28.1 ± 4.8α	Rhinorrhea	No adverse reactions
**Gelardi et al., 2013**	Italy	AR and vasomotor rhinitis	30 days	Mometasone furoate nasal spray (50μg/spray, 2 sprays/nostril once daily) Desloratadine (5mg, once daily) SH (9mg, twice daily)	39 (23)	21–63β	Mometasone furoate nasal spray (50μg/spray, 2 sprays/nostril once daily) Desloratadine (5mg, once daily) Sodium chloride (6mL, twice daily)	39 (21)	22–61β	Nasal Congestion, Rhinorrhea, Eosinophils, Neutrophils	Not reported
**Gelardi et al., 2016**	Italy	AR, NAR, and MR	4 weeks	intranasal mometasone furoate (1 puff/nostril, twice daily) rupatadine fumarate (1 tablet daily) isotonic saline solution (1 puff/nostril, twice daily) SH (1 cm per nostril in the afternoon)	48	Not reported	intranasal mometasone furoate (1 puff/nostril, twice daily) rupatadine fumarate (1 tablet daily) isotonic saline solution (1 puff/nostril, twice daily)	41	Not reported	Nasal Congestion, Rhinorrhea, Itching, Sneezing, Hyposmia	No adverse reactions
**Ocak et al., 2021**	Turkey	AR	30 days	Triamcinolone acetonide sprays (256 μg/day, 1 puff/nostril, once daily) Desloratadine (5mg, once daily) SH (9mg, twice daily)	32 (14)	34 (18–68) ^β^	Triamcinolone acetonide sprays (256 μg daily, 1 puff/nostril, once daily) Desloratadine (5mg, once daily) Isotonic saline (9mg, twice daily)	33 (13)	36 (18–61) ^β^	Mucociliary clearance	No adverse reactions
**Savietto et al., 2020**	Italy	CRSsNP	30 days	SH (5mg, twice daily)	15	Not reported	Isotonic saline solution (5mg, twice daily)	15	Not reported	Nasal Congestion, Rhinorrhea, Eosinophils, Neutrophils, Nasal Endoscopy Scoring, Quality of life, Hyposmia	No adverse reactions
**Thieme et al., 2020**	Germany	Dry nose symptoms	4 weeks	SH (1–2 sprays/nostril)/Hyaluronic acid plus dexpanthenol (1–2 sprays/nostril)	79 (41)/ 80 (25)	54.15 ± 17.03^α^/50.60 ± 18.98^α^	Isotonic saline (1–2 sprays/nostril)	80 (31)	50.27 ± 19.7^α^	Nasal Congestion, Rhinorrhea, Rhinitis, Itching, Sneezing, Hyposmia	Cephalgia

CRS, chronic rhinosinusitis; CRSwNP, chronic rhinosinusitis with nasal polyposis; CRSsNP, chronic rhinosinusitis without nasal polyposis; ARS, acute rhinosinusitis; NAR, nonallergic rhinitis; MR: mixed rhinitis; SH, sodium hyaluronate; α, Mean age ± SD; β, Mean age (range); *, median (IQR).

In the published article, there was an error. The authors found that the occurrence of cephalgia was possibly related the use of nasal spray (Thieme et al., 2020), however, this was omitted in the meta-analysis.

A correction has been made to **3 Results**, *3.4 Outcomes*, 3.4.4 Adverse events, Paragraph Number 1. The sentences previously stated:

“These studies (Cassandro et al., 2015; Ercan et al., 2022) reporting adverse events included nasal burning, headaches, throat irritation, upper respiratory tract infections, epistaxis, and nasal irritation. One trial (Cassandro et al., 2015) reported no difference in the incidence of adverse events between the HA and control groups, and the other trial (Ercan et al., 2022) showed only mild adverse events in the control group.”

The corrected sentence appears below:

“These studies (Cassandro et al., 2015; Ercan et al., 2022) reported adverse events including nasal burning, headache, throat irritation, upper respiratory tract infection, epistaxis, and nasal irritation. One patient reported thrice about the occurrence of cephalgia in hyaluronic acid plus dexpanthenol group, rated as possibly related to the application of the nasal spray (Thieme et al., 2020). One trial (Cassandro et al., 2015) reported no difference in the incidence of adverse events between the HA and control groups, and the other trial (Ercan et al., 2022) showed only mild adverse events in the control group.”

In the published article, there was an error in Table 1. In the final row, “α” was not in superscript. The corrected table and its caption appear above.

The authors apologize for these errors and state that this does not change the scientific conclusions of the article in any way. The original article has been updated.

