# Supplementary material for: Efficacy of hyaluronic acid in the treatment of nasal inflammatory diseases: a systematic review and meta-analysis
Source: Front Pharmacol. 2024 Feb 7;15:1350063. doi: 10.3389/fphar.2024.1350063 (PMC10879391; doi:10.3389/fphar.2024.1350063)
Supplement: Supplementary file 1 [file Table1.DOCX]

| **Source** | **Country** | **Type of patients** | **Duration** | **Intervention** | | | **Control** | | | **Outcomes** | **Adverse event** |
| --- | --- | --- | --- | --- | --- | --- | --- | --- | --- | --- | --- |
|  |  |  |  | **Intervention method（Dose）** | **Population**  **（Male）** | **Mean age** | **Intervention method（Dose）** | **Population**  **（Male）** | **Mean age** |  |  |
| Cantone et.al 2016 | Italy | CRSwNP | 3 months | Mometasone furoate nasal spray (200μg, once daily)  SH plus saline solution (9mg, twice daily) | 40 | 56.9±5.6^α^ | Mometasone furoate nasal spray (200μg, once daily)  Saline solution (5ml, twice daily) | 40 | 56.8±4.4^α^ | Nasal Congestion, Rhinorrhea, Nasal Endoscopy Scoring, Quality of life | No adverse reactions |
| Casale et.al 2014 | Italy | CRS | 3 months | SH plus saline solution (9mg, twice/day) | 21(13) | 44(30-63) ^β^ | Saline solution (5ml, twice/day) | 18(10) | 38(34-58) ^β^ | Rhinitis | No adverse reactions |
| Cassandro et.al 2015 | Italy | CRSwNP | 3 months | SH plus saline (9mg, twice daily) | 20(12) | 38.75±13.08^α^ | Saline (5ml, twice daily) | 20 (11) | 38.6±13.06^α^ | Rhinitis, Mucociliary clearance, Nasal Endoscopy Scoring, Rhinomanometry | Headache, Throat irritation, Upper respiratory infection, Epistaxis, Nasal burning |
|  |  |  |  | Mometasone furoate nasal sprays (200μg, twice daily)  SH (9mg, twice daily) | 20(12) | 38.85±13.31^α^ | Mometasone furoate nasal sprays (200μg, twice daily) | 20(10) | 38.4±12.7^α^ |  |  |
| Ciofalo et.al 2017 | Italy | ARS | 30 days | Levofloxacin (500mg, 10days)  Prednisone (50mg, 8days; 25mg, 4days; 12.5mg, 4days)  SH plus saline solution (6ml, twice daily) | 24(12) | 44(38-50) * | Levofloxacin (500mg, 10days)  Prednisone (50mg, 8days; 25mg, 4days; 12.5mg, 4days)  Saline solution (6ml, twice daily) | 24(14) | 43(35-55) * | Nasal Congestion, Rhinorrhea, Eosinophils, Neutrophils, mucociliary clearance, Hyposmia | Not reported |
| Ercan et. al 2022 | Turkey | AR in children | 28 days | Nasal fluticasone furoate (1 puff/nostril, once daily)  SH (twice daily) | 26(18) | 8.38±1.89^α^ | Nasal fluticasone furoate (1 puff/nostril, once daily)  Saline solution (twice daily) | 24(12) | 8.5±1.31^α^ | Nasal Congestion, Rhinorrhea, Rhinitis, Itching, Sneezing, Eosinophils, Quality of life, Rhinomanometry | Nasal irritation and burning sensation |
|  |  |  |  | Nasal fluticasone furoate (1 puff/nostril, once daily)  SH (twice daily) | 26(18) | 8.38±1.89^α^ | Nasal fluticasone furoate (1 puff/nostril, once daily) | 26(18) | 8.69±1.7^α^ |  |  |
| Favilli et.al 2019 | Italy | Pregnancy Rhinitis | until delivery | SH (9mg/vial; 2 vials daily for 14 days, followed by 15 days of interruption of therapy; subsequently 1 vial daily for 10 and 15 days of interruption of therapy; lastly 1 vial daily for 10 days) | 28 | 31.6±5.5^α^ | Not receive any treatment | 27 | 28.1±4.8^α^ | Rhinorrhea | No adverse reactions |
| Gelardi et.al 2013 | Italy | AR and vasomotor rhinitis | 30 days | Mometasone furoate nasal spray (50μg/spray, 2 sprays/nostril once daily)  Desloratadine (5mg, once daily)  SH (9mg, twice daily) | 39(23) | 21-63^β^ | Mometasone furoate nasal spray (50μg/spray, 2 sprays/nostril once daily)  Desloratadine (5mg, once daily)  Sodium chloride (6ml, twice daily) | 39(21) | 22-61^β^ | Nasal Congestion, Rhinorrhea, Eosinophils, Neutrophils | Not reported |
| Gelardi et.al 2016 | Italy | AR, NAR, and MR | 4 weeks | intranasal mometasone furoate (1 puff/nostril, twice daily)  rupatadine fumarate (1 tablet daily)  isotonic saline solution (1 puff/nostril, twice daily)  SH (1 cm per nostril in the afternoon) | 48 | Not reported | intranasal mometasone furoate (1 puff/nostril, twice daily)  rupatadine fumarate (1 tablet daily)  isotonic saline solution (1 puff/nostril, twice daily) | 41 | Not reported | Nasal Congestion, Rhinorrhea, Itching, Sneezing, Hyposmia | No adverse reactions |
| Ocak et.al 2021 | Turkey | AR | 30 days | Triamcinolone acetonide sprays (256μg/day, 1 puff/nostril, once daily)  Desloratadine (5mg, once daily)  SH (9mg, twice daily) | 32(14) | 34(18-68) ^β^ | Triamcinolone acetonide sprays (256μg daily, 1 puff/nostril, once daily)  Desloratadine (5mg, once daily)  Isotonic saline (9mg, twice daily) | 33(13) | 36(18-61) ^β^ | Mucociliary clearance | No adverse reactions |
| Savietto et.al 2020 | Italy | CRSsNP | 30 days | SH (5mg, twice daily) | 15 | Not reported | Isotonic saline solution (5mg, twice daily) | 15 | Not reported | Nasal Congestion, Rhinorrhea, Eosinophils, Neutrophils, Nasal Endoscopy Scoring, Quality of life, Hyposmia | No adverse reactions |
| Thieme et.al 2020 | Germany | dry nose symptoms | 4 weeks | SH (1–2 sprays/nostril) | 79(41) | 54.15±17.03^α^ | Isotonic saline (1–2 sprays/nostril) | 80(31) | 50.27±19.7^α^ | Nasal Congestion, Rhinorrhea, Rhinitis, Itching, Sneezing, Hyposmia | Not reported |

CRS: chronic rhinosinusitis; CRSwNP: chronic rhinosinusitis with nasal polyposis; CRSsNP: chronic rhinosinusitis without nasal polyposis; ARS: acute rhinosinusitis; NAR: nonallergic rhinitis; MR: mixed rhinitis; SH: sodium hyaluronate; α: Mean age ± SD; β: Mean age (range); *: median (IQR)
