# Supplementary material for: Efficacy of hyaluronic acid in the treatment of nasal inflammatory diseases: a systematic review and meta-analysis
Source: Front Pharmacol. 2024 Feb 7;15:1350063. doi: 10.3389/fphar.2024.1350063 (PMC10879391; doi:10.3389/fphar.2024.1350063)
Supplement: Supplementary file 2 [file Table2.DOCX]

**Efficacy of Hyaluronic Acid in the Treatment of Nasal Inflammatory Diseases: A Systematic Review and Meta-analysis**

Supplementary Online Content

eTable 1. Search strategy in PubMed

| PubMed | | |
| --- | --- | --- |
| 1 | "Rhinitis, Allergic"[Mesh] | **23487** |
| 2 | ((Allergic Rhinitis[Title/Abstract]) OR (Rhinitides, Allergic[Title/Abstract])) OR (Allergic Rhinitides[Title/Abstract]) | **27222** |
| 3 | "Sinusitis"[Mesh] | **23010** |
| 4 | ((((Sinus Infection[Title/Abstract]) OR (Infections, Sinus[Title/Abstract])) OR (Infection, Sinus[Title/Abstract])) OR (Sinus Infections[Title/Abstract])) OR (Sinusitides[Title/Abstract]) | **832** |
| 5 | "Rhinitis" [Mesh] | **38135** |
| 6 | ((((("Rhinitis"[Mesh]) OR (Rhinitides[Title/Abstract])) OR (Nasal Catarrh[Title/Abstract])) OR (Catarrh, Nasal[Title/Abstract])) OR (Catarrhs, Nasal[Title/Abstract])) OR (Nasal Catarrhs[Title/Abstract]) | 38742 |
| 7 | "Hyaluronic Acid"[Mesh] | 25395 |
| 8 | ((((((((((((Healon[Title/Abstract]) OR (Amvisc[Title/Abstract])) OR (Hyaluronate Sodium[Title/Abstract])) OR (Hyaluronate, Sodium[Title/Abstract])) OR (Sodium Hyaluronate[Title/Abstract])) OR (Luronit[Title/Abstract])) OR (Hyvisc[Title/Abstract])) OR (Hyaluronan[Title/Abstract])) OR (Etamucine[Title/Abstract])) OR (Biolon[Title/Abstract])) OR (Vitrax, Amo[Title/Abstract])) OR (Amo Vitrax[Title/Abstract])) OR (Acid, Hyaluronic[Title/Abstract]) | 12773 |
| 9 | Randomized controlled trial OR Controlled clinical trial OR Randomized OR Placebo OR Randomly | 1790637 |
| 10 | (1 OR 2) and (3 OR 4) and (5 OR 6) and (7 OR 8) and (9) | 38 |

eTable 2

| **Unique ID** | **Reviewer** | **Study ID** | **Reference** | **Experimental** | **Comparator** | **Outcome** | **Result** | **Aim** | **Weight** | **Randomization process** | **Deviations from intended interventions** | **Mising outcome data** | **Measurement of the outcome** | **Selection of the reported result** | **Overall Bias** |
| --- | --- | --- | --- | --- | --- | --- | --- | --- | --- | --- | --- | --- | --- | --- | --- |
| Cantonese 2016 | Wang | Cantonese 2016 | 10.2500/ajra.2016.30.4344 | HA | Saline solution | Nasal Congestion score, Rhinorrhea score | | assignment to intervention (the 'intention-to-treat' effect) | 1 | Low | Low | Low | Low | Low | Low |
| Casale 2014 | Wang | Casale 2014 | 10.2500/ajra.2014.28.4045 | HA | Saline solution | Rhinitis score |  | assignment to intervention (the 'intention-to-treat' effect) | 1 | High | Some concerns | Low | Low | Low | High |
| Cassandro 2015 | Wang | Cassandro 2015 | 10.1007/s12070-014-0766-7 | HA | Saline solution | Rhinitis score, Mucociliary clearance time, Nasal Endoscopy Scoring, rhinomanometry | | assignment to intervention (the 'intention-to-treat' effect) | 1 | High | Some concerns | Low | Low | Low | High |
| Ciofalo 2017 | Wang | Ciofalo 2017 | 2017; 21: 4411-4418 | HA | Saline solution | Nasal Congestion score, Rhinorrhea score, Eosinophils, Neutrophils, mucociliary clearance time, Hyposmia | | assignment to intervention (the 'intention-to-treat' effect) | 1 | Low | Low | Low | Low | Low | Low |
| Ercan 2022 | Wang | Ercan 2022 | 10.1007/s00405-021-07073-0 | HA | Saline solution | Nasal Congestion score, Rhinorrhea score, Rhinitis score, Itching score, Sneezing score, Eosinophils, Quality of life score, rhinomanometry | | assignment to intervention (the 'intention-to-treat' effect) | 1 | High | Some concerns | Low | Low | Low | High |
| Favilli 2019 | Wang | Favilli 2019 | 10.1159/000493137 | HA | Not receive any treatment | Rhinorrhea score | | assignment to intervention (the 'intention-to-treat' effect) | 1 | High | Some concerns | Low | Low | Low | High |
| Gelardi 2013 | Wang | Gelardi 2013 | 10.1002/alr.21193 | HA | Saline solution | Nasal Congestion score, Rhinorrhea score, Eosinophils, Neutrophils | | assignment to intervention (the 'intention-to-treat' effect) | 1 | Low | Low | Low | Low | Low | Low |
| Gelardi 2016 | Wang | Gelardi 2016 | Vol. 30, no. 1, 255-262 (2016) | HA | Saline solution | Nasal Congestion score, Rhinorrhea score, Itching score, Sneezing score, Hyposmia | | assignment to intervention (the 'intention-to-treat' effect) | 1 | High | Some concerns | Low | Low | Low | High |
| Ocak 2021 | Wang | Ocak 2021 | 10.1017/S0022215121000967 | HA | Saline solution | Mucociliary clearance time | | assignment to intervention (the 'intention-to-treat' effect) | 1 | Low | Some concerns | Low | Low | Low | Some concerns |
| Savietto 2020 | Wang | Savietto 2020 | 10.1016/j.amjoto.2020.102502 | HA | Saline solution | Nasal Congestion score, Rhinorrhea score, Eosinophils, Neutrophils, Nasal Endoscopy Scoring, Quality of life score, Hyposmia | | assignment to intervention (the 'intention-to-treat' effect) | 1 | Low | Low | Low | Low | Low | Low |
| Thieme 2020 | Wang | Thieme 2020 | 10.1016/j.anl.2020.01.008 | HA | Saline solution | Nasal Congestion score, Rhinorrhea score, Rhinitis score, Itching score, Sneezing score, Hyposmia | | assignment to intervention (the 'intention-to-treat' effect) | 1 | Low | Low | Low | Low | Low | Low |

HA: hyaluronic a


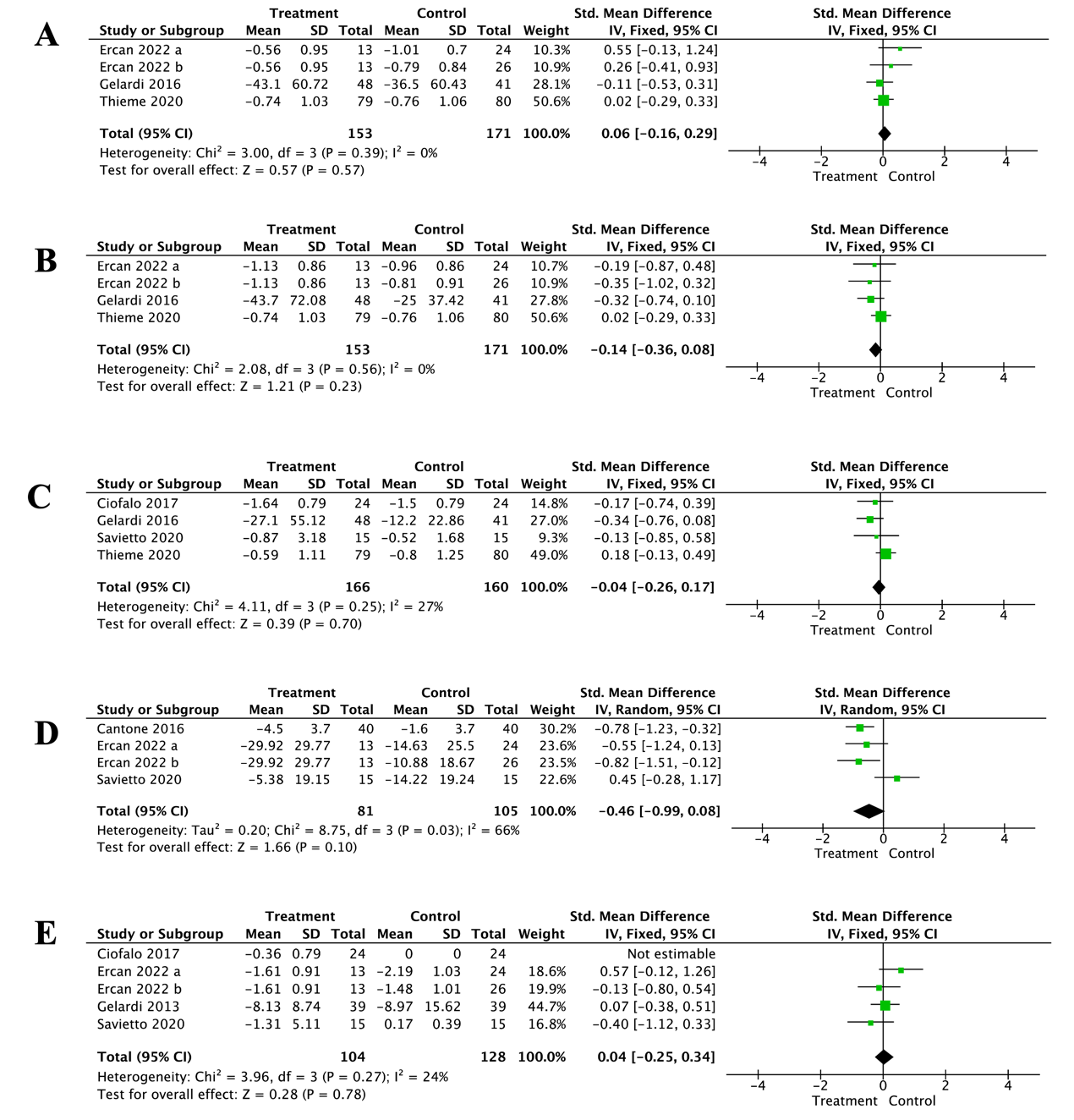
Fig. 1: Forest plot for nasal itching (A), sneezing (B), hyposmia (C), quality of life score (D), and eosinophils (E).


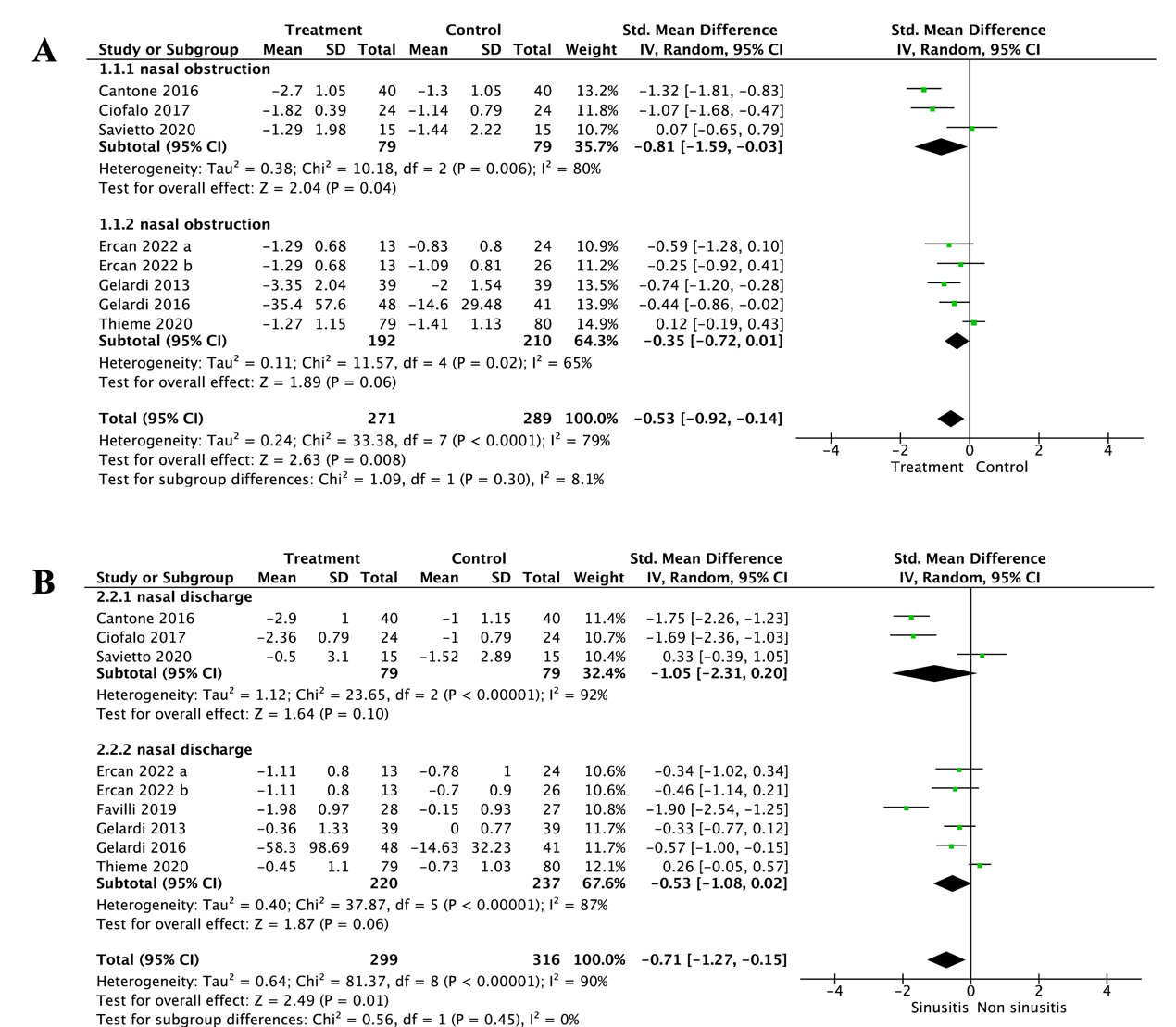
Fig. 2: Forest plot for nasal obstruction (A) and rhinorrhea (B) in sinusitis group or non-sinusitis group.


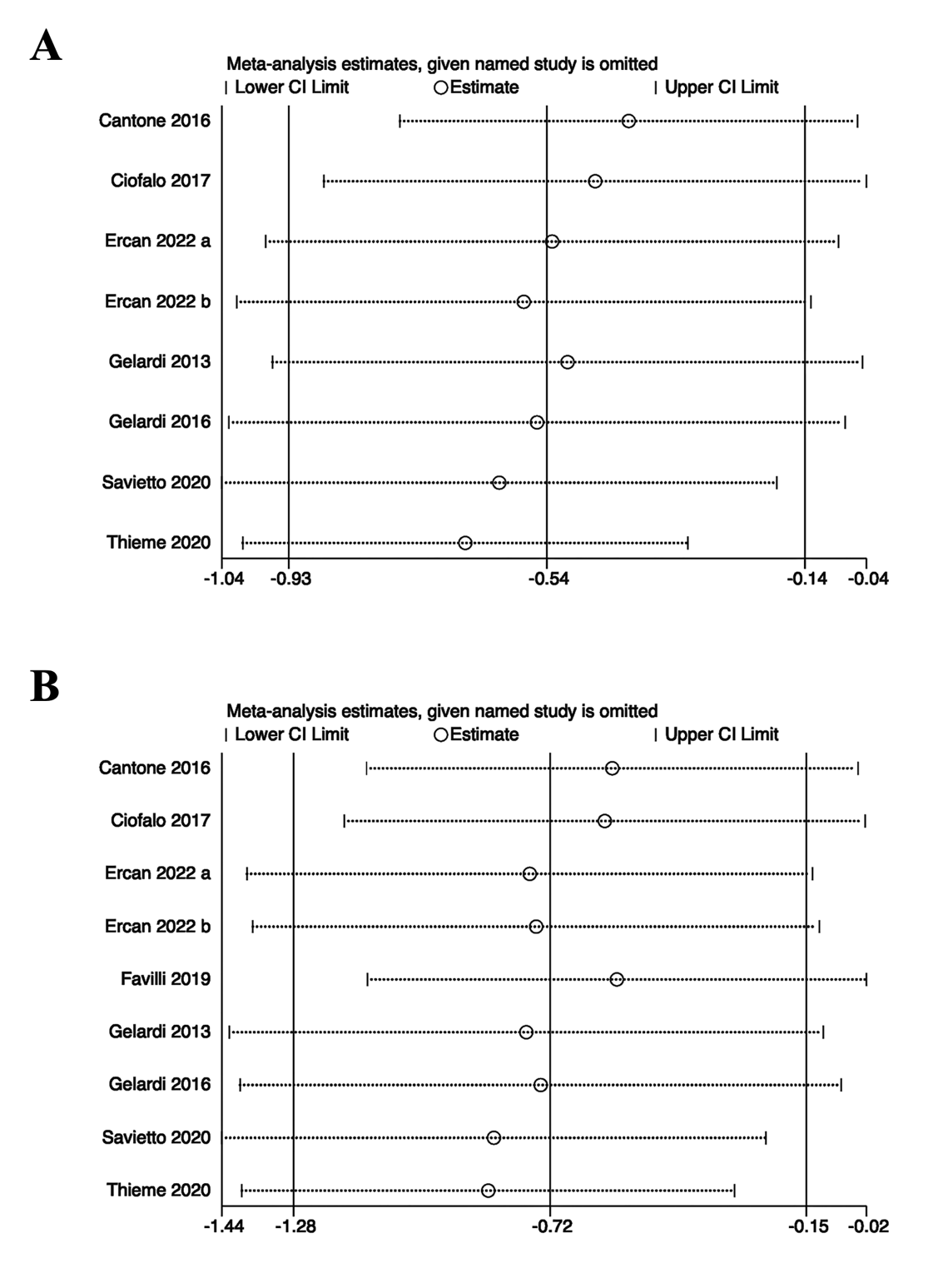
Fig. 3 Sensitivity analysis for nasal obstruction (A) and rhinorrhea (B).
